# Closing the Gender Gap in Fragile X Syndrome: Review of Females with Fragile X Syndrome and Preliminary Research Findings

**DOI:** 10.3390/brainsci9010011

**Published:** 2019-01-12

**Authors:** Kristi L. Bartholomay, Cindy H. Lee, Jennifer L. Bruno, Amy A. Lightbody, Allan L. Reiss

**Affiliations:** 1 Department of Psychiatry and Behavioral Sciences, Stanford University, Stanford, CA 94305, USA; kbarthol@stanford.edu (K.L.B.); jenbruno@stanford.edu (J.L.B.) aal@stanford.edu (A.A.L.); areiss1@stanford.edu (A.L.R.); 2 Department of Radiology, Stanford University, Stanford, CA 94305, USA; 3 Department of Pediatrics, Stanford University, Stanford, CA 94305, USA

**Keywords:** fragile X syndrome, X chromosome, females, FMR1, anxiety, avoidance, cognition, behavior, brain

## Abstract

Fragile X syndrome (FXS) is a genetic condition known to increase the risk of cognitive impairment and socio-emotional challenges in affected males and females. To date, the vast majority of research on FXS has predominantly targeted males, who usually exhibit greater cognitive impairment compared to females. Due to their typically milder phenotype, females may have more potential to attain a higher level of independence and quality of life than their male counterparts. However, the constellation of cognitive, behavioral, and, particularly, socio-emotional challenges present in many females with FXS often preclude them from achieving their full potential. It is, therefore, critical that more research specifically focuses on females with FXS to elucidate the role of genetic, environmental, and socio-emotional factors on outcome in this often-overlooked population.

## 1. Introduction to Fragile X Syndrome

Fragile X syndrome (FXS) is a genetic condition that is commonly cited as the leading heritable cause of autism and intellectual disability. Though estimates vary widely, FXS is expected to occur in one in every 2500 to 7000 males and one in 2500 to 11,000 females [1,2,3]. Males with FXS tend to exhibit more cognitive and behavioral problems relative to females with FXS and, therefore, tend to come to the attention of medical and mental health providers more frequently than females. 

The majority of males with FXS meet criteria for severe intellectual disability. The most common behavioral features include attention deficits and hyperactivity, anxiety, and symptoms of autism spectrum disorder [4]. In contrast, the phenotype in females with FXS is generally less severe and more frequently associated with learning disabilities, socio-emotional difficulties, and mental health issues [5]. This difference in symptom presentation and severity has led, historically, to females receiving a diagnosis only after a close male relative is diagnosed, leaving many girls and women with this condition unidentified.

To date, the vast majority of clinical intervention and research funding for FXS has focused predominately on affected males. The relative scarcity of research into the unique phenotype of females with FXS represents a significant gap in the field; however, it also represents a potent target for ongoing research and intervention. Given their typically milder phenotype, females may have more potential to attain a higher level of independence and quality of life than their male counterparts, who will likely need a moderate or greater level of support throughout their lives. However, lack of specified treatments and resources specifically targeting girls with this condition results in outcomes that are often no better than those of their more affected male counterparts. Increased emphasis on research in females with FXS and the development of new treatments is, therefore, paramount. 

## 2. Genetics

### 2.1. Pattern of Inheritance of Fragile X Syndrome

FXS is a trinucleotide repeat disorder resulting from an expanded CGG repeat in the untranslated region of the fragile X mental retardation 1 (*FMR1*) gene. While the unaffected gene carried by the majority of the population contains fewer than 55 repeats, individuals with full mutation FXS typically have greater than 200 repeats in this region. Individuals who fall in the intermediate range with 55 to 200 repeats are classified as having the fragile X premutation, which is associated with its own distinct phenotype [6,7]. This paper will focus exclusively on full mutation (greater than 200 repeats) FXS. 

In full mutation FXS, the repeat region is hypermethylated, resulting in transcriptional silencing of the *FMR1* gene, and, therefore, reduced production of the encoded protein, the fragile X mental retardation protein (FMRP). FMRP is an RNA binding protein which plays a key role in regulating local protein synthesis as well as a number of other functions [8,9]. In the absence of FMRP, synthesis of target proteins is dysregulated, resulting in the FXS phenotype. However, even among people with fragile X full mutation, there is significant variability in the phenotypic presentation of the syndrome [10].

### 2.2. Genetic Foundation of Sex Differences

The *FMR1* gene is located on the X chromosome, leading to the significant sex differences observed in the FXS phenotype. Males have only one X chromosome. Thus, if they inherit the X chromosome with the *FMR1* full mutation from their mother, 100% of their X chromosomes are potentially “affected” [11]. Females, however, have two X chromosomes. If they inherit the X chromosome with the *FMR1* full mutation from their mother and the unaffected X chromosome from their father, only 50% of their X chromosomes are potentially affected. The second, “unaffected” X chromosome allows the production of some FMRP, but the dosage is generally not sufficient to restore full FMRP function in most heterozygous females [12]. This is the basis for the typically milder, but still affected, phenotype seen in females with FXS.

**Figure 1:**
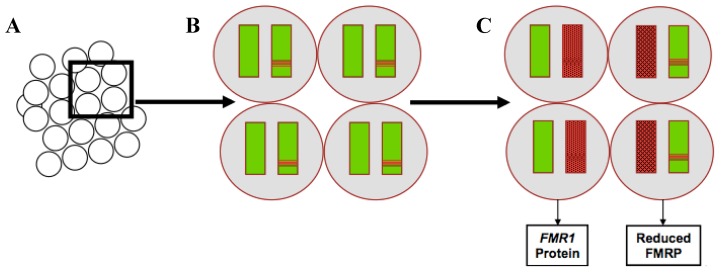
X-chromosome inactivation in females with fragile X syndrome and corresponding production of fragile X mental retardation 1 (*FMR1*) protein in each cell. (**A**) Group of progenitor cells. (**B**) Each cell contains two X chromosomes. The unaffected chromosomes are shown in solid green, and affected chromosomes are depicted with a red band. (**C**) One of the two chromosomes in each cell will be silenced at random indicated with red shading. In cells where the affected chromosome is silenced, there is normal production of the *FMR1* protein. In cells where the unaffected chromosome is silenced, there is reduced production of the *FMR1* protein. *FMR1*: fragile X mental retardation 1; FMRP: fragile X mental retardation protein.

The variation in phenotype among females with full mutation FXS can, at least in part, be attributed to a phenomenon known as X inactivation [11,13]. X inactivation is thought to occur so that females have approximately equal X chromosome gene dosage despite having twice as many X chromosomes (known as “dosage compensation” [14]). During female embryonic development, most of the genes on one X chromosome in each cell are randomly silenced, resulting in approximately half the cells in the body expressing the genes from each X chromosome. It is believed that only a small number of embryonic progenitor cells will go on to form the brain, so the ratio of cells that have the affected X active to silenced is thought to significantly affect the level of FMRP expression in the developing central nervous system (Figure 1). The variance of this ratio likely contributes to the widely variable phenotype for females with FXS [11].

## 3. The Fragile X Phenotype in Females

Inheriting the *FMR1* full mutation does not directly correspond to the development of the fragile X syndrome phenotype, but rather represents genetic risk for a particular set of cognitive, socio-emotional, and behavioral outcomes [15]. Phenotypic signs and symptoms will be expressed differently as a result of X inactivation, other genetic factors, and environmental influences. Many females with FXS meet criteria for one or more Diagnostic and Statistical Manual of Mental Disorders-5 (DSM-5) diagnoses—generalized anxiety disorder (GAD), social anxiety disorder (SAD), math learning disability (LD), intellectual disability (ID), autism spectrum disorder (ASD), and attention deficit hyperactivity disorder (ADHD) to name a few. These diagnoses are useful insofar as they enable the individual to receive necessary services and accommodations. DSM-5 diagnoses can also facilitate communication among mental health professionals. 

The phenotypic profile for males with FXS has been relatively well established and includes moderate to severe intellectual disability by the time affected individuals reach adolescence or adulthood, high co-occurrence of autism spectrum disorder symptoms, self-injurious and aggressive behaviors, and attention deficits [4]. Very few studies have focused exclusively on females with the *FMR1* full mutation. The female phenotype is less predictable and often less severe, at least with respect to general cognitive effects. However, females with FXS do not necessarily achieve better outcomes than their male counterparts, and the socio-emotional burden on affected girls, women, and their families may still be equal to that of affected males [16,17]. It is, therefore, crucial that females be equally represented in research to establish a fundamental understanding of sex differences in FXS, the neural foundations for these differences, and potential targets for intervention to improve the quality of life and health outcomes for this often-overlooked population. 

### 3.1. Cognitive Effects

The cognitive effect of FXS in females is variable, ranging from moderate intellectual disability to an average or above average cognitive profile [18]. Females with FXS often exhibit challenges with executive functioning and impaired spatial reasoning skills, with relative strengths in verbal skills [10,19,20,21]. Although females with FXS consistently show milder deficits in both cognitive and academic function than males with FXS, on average their scores fall below those of typically developing peers [10,16,22]. The discrepancy in cognitive function between girls with FXS and their typically developing peers is larger for IQ than for academic achievement scores [23]. 

A previous study by our group demonstrated that executive functioning scores declined over time in girls with FXS while other aspects of intelligence, such as verbal fluency and spatial ability, remained stable throughout childhood [24]. It is likely that these declines are not due to the loss of skills, but rather to a slowing rate of acquisition of new skills compared to typically developing peers, as well as the increasingly strenuous cognitive demands placed on children as they age. A recent longitudinal study on girls with FXS suggested that, while fluid intelligence was predicted only by biological or genetic factors, crystalized intelligence may be related to maternal mental health and perceived closeness of mother–child relationship [13]. Taken together, these studies suggest a vulnerable time frame in which girls with FXS are prone to decline in cognitive scores, and the importance of protective factors, such as an enriched environment, that may help mitigate this decline. It is, therefore, critical that research focus on understanding vulnerable timeframes in the cognitive development of girls with FXS, while simultaneously developing medical, psychological, and educational interventions to facilitate improved long-term outcomes.

### 3.2. Socio-Emotional Effects

Anxiety, avoidance, and arousal (AAA) represent three key behaviors exhibited in response to acute, potential, and sustained threat, respectively. These behaviors represent typical responses to aversive or dangerous stimuli. However, existing evidence suggests that the dysregulation of these systems can result in clinical manifestation of disorders of emotion and affect including anxiety and depression [25]. Such disorders are commonly cited as primary clinical concerns for females with FXS [4] and may be, at least in part, responsible for the often-large disparity between actual outcomes and those predicted by IQ and life skill proficiency [4,17]. Reducing AAA could improve quality of life and outcomes for girls and women with FXS. Thus, AAA represent critical targets for intervention.

In one of the first studies looking specifically at girls with FXS, Freund et al. found that females with FXS were more vulnerable to social anxiety, social avoidance, withdrawal, and depression than their IQ and age-matched peers. Furthermore, affected females showed deficits in interpersonal and social skills compared to peers who did not carry the fragile X full mutation [21]. In another study, parental report indicated that 56% of girls and women with FXS had received treatment for and/or diagnosis of an anxiety disorder while 22% had received treatment for and/or diagnosis of depression [4]. Another study found that 51.4% of girls with FXS met diagnostic criteria for specific phobia, 39.5% met criteria for social phobia, and 25.3% met criteria for selective mutism. These rates are significantly higher than those of the general population or for individuals with intellectual disability alone [26]. 

When completing a social challenge, girls with FXS show more gaze aversion, task avoidance, and behavioral signs of distress than their typically developing siblings, and both boys and girls with FXS exhibit aberrant cortisol reactivity, and parasympathetic and sympathetic nervous system dysregulation compared to their same-sex typically developing siblings [27,28]. Further, Hartley et al. found that the largest predictor of independence for adult women with FXS was the ability to interact appropriately in social situations and that independence was inversely correlated with the presence of co-occurring anxiety disorders or depression [17].

Together, these studies support prior findings suggesting that anxiety experienced by girls with FXS can, in part, be attributed to impairment in social skills and social communication [29,30]. The impact of atypical development of social skills and communication in girls over time may lead to a wide range of anxiety symptoms or disorders. However, there is little understanding as to how this process occurs and how biological and environmental factors interact to affect the outcome. More research into the longitudinal profile of anxiety in girls with FXS is necessary to facilitate such an understanding and for the eventual development of more effective, disorder-specific interventions to improve social and communication skills. In particular, interventions implemented before the development of anxiety symptoms and disorders in girls with FXS hold particular promise for achieving a more optimal long-term outcome. 

### 3.3. Adaptive Behavior and Independent Living

Both males and females with FXS experience significant difficulty in the acquisition of adaptive behavior skills compared to their typically developing peers and struggle to reach independence, often continuing to live with their parents or in an assisted living environment once they reach adulthood [17]. Adaptive behaviors, or daily living skills, are a broad group of skills and abilities accumulated throughout childhood and into adulthood which allow an individual to function in their daily environment, including skills like verbal and written communication, routine self-care, and social skills. 

Several studies have investigated the acquisition of adaptive behavior in children with FXS. In one such study, parents of children with FXS were interviewed about their child’s adaptive behavior skills. Consistent with previous studies [31], females with FXS typically scored higher on adaptive behavior than males with FXS at all ages. However, their adaptive behavior skills appeared to decline throughout childhood. These declines were most pronounced in the domain of communication, suggesting that as girls with FXS age they fall further behind peers in their verbal and written communication skills [32]. This does not necessarily indicate that females with FXS are losing skills during childhood, but rather that they are not gaining skills at the same rate as their typically developing peers. Another study demonstrated that both IQ and quality of home environment are predictive of adaptive behavior in boys with FXS and in the unaffected siblings of children with FXS; however, only IQ appears to be predictive of adaptive behavior outcomes in girls with FXS [15]. This suggests that boys may have more adaptive behavior skills than their IQ would predict due to support and accommodations in the home and school environments allowing them to succeed, while girls with FXS may not be receiving sufficient support or intervention, or their difficulties may not be identified early enough to implement effective strategies. 

Various studies have also assessed the functional and independent living skills of adults with FXS. In one study, parents of children with FXS rated their level of independence in various daily skills. By adulthood, the majority of both males and females with FXS were able to independently complete most tasks, and females acquired independent living skills significantly faster and reached a higher level of proficiency than their male counterparts [33]. In another study, parents were asked to rate the level of independence of their adult children in these types of skills as well as rate their general level of independence across various domains. In this study, for males with FXS, level of independence was best predicted by proficiency in daily living skills. However, the strongest predictor of independence for females with FXS was the ability to interact appropriately in social settings, an area of significant challenge for affected individuals. Less than half of women with FXS reached very high or high levels of independence, even though they had significantly more functional skills than their male counterparts [17].

These studies suggest that, although females with FXS have significantly higher proficiency in daily living skills both in childhood and adulthood than their male counterparts, there is not a corresponding increase in their actual levels of independence in adult life. This disparity between girls’ potential, as represented by their IQ and functional skills, and their resulting level of independence represents a critical target for research and intervention. To ensure girls with FXS are reaching their maximum potential, it must first be better understood how, when, and why they are falling short of reaching independence, and interventions must directly target these timeframes and skills to substantively improve outcomes. 

### 3.4. Barriers to Positive Outcomes

Factors other than genetics play an important role in determining outcomes for females with FXS. Such factors include severity of social-emotional symptoms, home/family environment, parental mental health, and parenting style [24], and likely account for some of the variability in outcome seen in girls with FXS. These factors also represent important potential targets of intervention. 

Several studies indicate that independence and quality of life for females with FXS are heavily influenced by the DSM-5 disorders for which females show symptoms or reach diagnostic criteria [4,16,30]. The severity of autism spectrum disorder symptomology is significantly associated with independent living outcomes for individuals with FXS, and symptoms of affective disorders, such as anxiety and depression, represent a barrier to achieving independence for females with FXS specifically [17,34]. A national survey of parents of children with FXS demonstrated that ability to adapt to changing situations, thinking and reasoning skills, and perceived quality of life were all inversely correlated with the number of DSM diagnoses for which the child met criteria. The most common diagnoses among females with FXS were attention deficit, anxiety, hyperactivity, and depression [4]. FXS itself cannot be prevented or treated, so it is crucial that the symptoms of these conditions are identified early. Early detection can lead to more targeted treatment and ultimately improve the outcomes and lives of girls with FXS and their families. 

The quality of the home environment also plays a role in determining the outcome for both boys and girls with FXS. One study found that the home environment influences behavioral outcomes for girls with FXS. Specifically, reported increases in parent psychopathology were correlated with an increase of child anxiety and depression, while increased efficacy of services was correlated with a decrease in thought and attention problems for the individual with FXS [35]. Another study found that increased quality of home environment corresponded with an increase in verbal IQ as well as an increase in freedom from distractibility [36], and a recent study published exclusively on girls with FXS demonstrated that lower IQ and increased social aversion could be predicted by higher levels of maternal distress and reduced perceived closeness of parent–child relationships [13]. These studies emphasize the importance of providing resources and support for families of girls with FXS to create home, family, and educational environments that promote their development and optimize behavioral, cognitive, and quality of life outcomes. 

To this end, our research team is implementing a prospective longitudinal study of girls with FXS. Highlights from some preliminary findings in a small sample set are presented below.

## 4. Methods

### 4.1. Participants

The preliminary cohort presented here consists of 21 girls between the ages of 6 to 14 (mean age = 10.53). Participants were recruited through various FXS communities including regional fragile X organizations, the Fragile X Clinical and Research Consortium, and the Fragile X Online Registry With Accessible Research Database, electronic media including website and social media announcements, and with the help of the National Fragile X Foundation. All participants were diagnosed by an appropriate molecular genetic test as having more than 200 CGG repeats in the *FMR1* gene with documentation of previous testing provided by caregivers at enrollment. All participants and their caregivers were native English speakers. The data presented here represent a partial sample from the first year and a half of the current five-year longitudinal study and will constitute approximately 40% of our final overall cohort. The research team is still actively recruiting and seeing participants.

### 4.2. Measurements/Procedures

#### 4.2.1. Cognition and Academics

Cognition was assessed with the Differential Ability Scales, 2nd Edition (DAS-II), which provides subscales for verbal, nonverbal, and spatial reasoning ability and an overall composite score [37]. Academic skills were assessed with The Kaufman Test of Educational Achievement, Third Edition Brief Form (KTEA-3 Brief), which provides reading and math subscales and an overall achievement composite score [38].

#### 4.2.2. Child Behavior and Emotion

Participants’ caregivers completed the Social Responsiveness Scale-2 (SRS) [39]. The SRS addresses social awareness, social information processing, capacity for reciprocal social responses, social anxiety/avoidance, and characteristic autistic preoccupations/traits. 

#### 4.2.3. Adaptive Behavior and Functional Skills

Adaptive behavior and functional skills in this cohort were assessed utilizing the Vineland Adaptive Behavior Scales, Third Edition—Interview Form (VABS-III) which measures adaptive behavior across the domains of communication, daily living skills, and socialization and includes an overall adaptive behavior composite score [40]. Caregivers completed the VABS-III interview with a trained researcher during their research visit. 

### 4.3. Data Analyses

Exploratory analyses were conducted to assess differences between cognitive and achievement scores and correlations between domains of cognitive, adaptive and social function. Results with a *p* value ≤0.05 were considered significant.

### 4.4. Preliminary Findings

#### 4.4.1. Cognition and Academics

**Figure 2:**
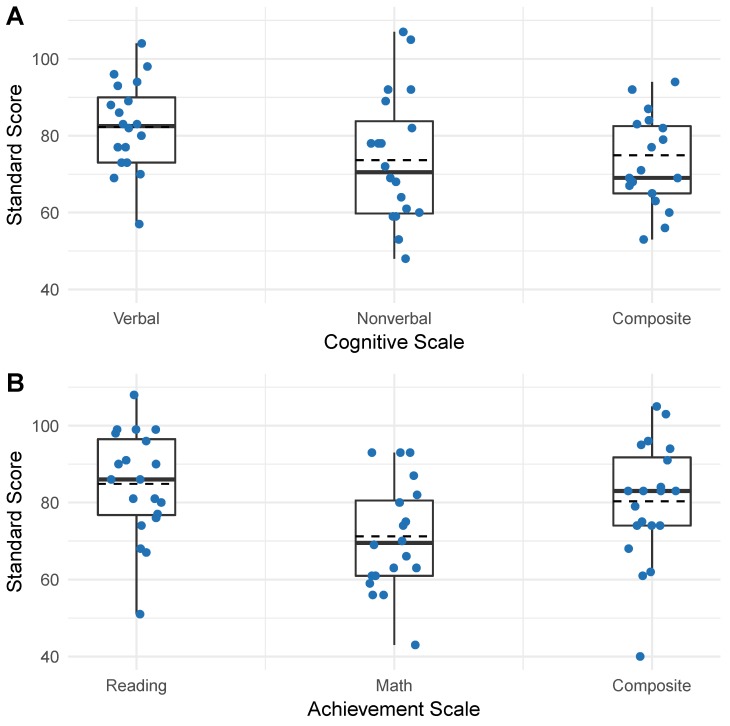
Cognitive and achievement score profiles. Each dot indicates a participant, solid horizontal line represents median, dashed horizontal line represents mean, box represents interquartile range, and vertical lines upper and lower extremes (excluding outliers). (**A**) Distribution of participant cognitive scores on verbal, nonverbal, and overall composite of the Differential Ability Scales, 2nd Edition (DAS-II). (**B**) Distribution of participant achievement scores on reading, math, and composite achievement on the Kaufman Test of Educational Achievement Third Edition Brief Form (KTEA-3 Brief).

The distributions of the cognitive and academic assessments are presented in Figure 2. In this preliminary cohort, girls with FXS performed significantly better on the verbal domain (*Mean* = 82.25, *SD* = 11.56) than on the nonverbal domain (*Mean* = 73.65, *SD* = 16.79); *t*(19) = 2.46, *p* = 0.012 or the overall composite (*Mean* = 74.90, *SD* = 14.77); *t*(19) = 3.10, *p* = 0.003 of the DAS-II (Figure 2A). Girls in this cohort also performed significantly better on the reading domain (*Mean* = 84.85, *SD* = 13.74) than on the math domain (*Mean* = 71.20, *SD* = 14.05); *t*(19) = 6.87, *p* = 0.000 or the overall composite (*Mean* = 80.35, *SD* = 15.50); *t*(19) = 3.07, *p* = 0.003 of the KTEA-Brief (Figure 2B). 

#### 4.4.2. Correlations between Cognition and Adaptive Behavior

**Figure 3:**
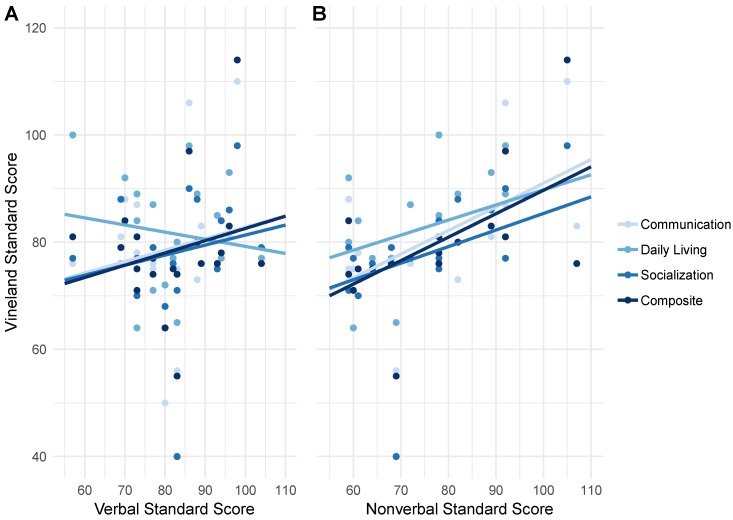
Correlations between cognition and adaptive behavior. (**A**) Correlations of the DAS-II verbal reasoning subscale with communication, daily living, and socialization subscales and overall adaptive behavior composite of the Vineland Adaptive Behavior Scales, Third Edition—Interview Form (VABS-III). (**B**) Correlations of the DAS-II nonverbal reasoning subscale with all domains of the VABS-III.

The verbal and nonverbal domains of the DAS-II are compared with corresponding performance on each subdomain of the VABS-III in Figure 3. Significant positive (Pearson) correlations were found between nonverbal reasoning ability and VABS-III communication *r*(18) = 0.58, *p* = 0.004, daily living skills r(18) = 0.59, *p* = 0.003, and overall composite *r*(18) = 0.58, *p* = 0.004 (Figure 3B) while significant correlations were not observed between verbal reasoning ability and any subdomains of the VABS-III (Figure 3A).

#### 4.4.3. Correlations between Social Skills and Adaptive Behavior

**Figure 4:**
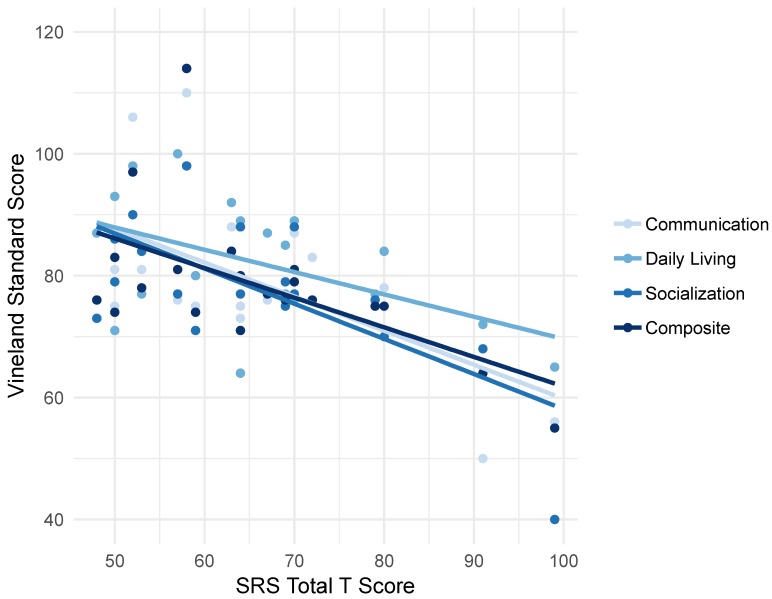
Correlations between social skills and adaptive behavior. Correlations between difficulties with social skills as measured by total score reported by caregivers on the Social Responsiveness Scale-2 (SRS) and the communication, daily living, and socialization subscales and overall adaptive behavior composite scores of the Vineland Adaptive Behavior Scales, Third Edition—Interview Form (VABS-III).

The associations between total score on the SRS-2 and corresponding scores on each VABS-III domain are presented in Figure 4. Increasing SRS scores (representing increasingly aberrant social skills), were correlated with decreasing VABS-III scores across all subdomains: communication *r*(18) = −0.62, *p* = 0.002, daily living skills *r*(18) = −0.45 *p* = 0.023, socialization *r*(18) = −0.747, *p* = 0.000, composite *r*(18) = −0.618, *p* = 0.002. 

## 5. Discussion

### 5.1. Cognition and Academics

Consistent with prior investigations of girls with FXS, the overall cognitive profile of children from our preliminary cohort was within the borderline/low average range [41]. The preliminary cohort presented here also scored significantly higher on the verbal subdomain than on the non-verbal subdomain of the DAS-II cognitive assessment (Figure 1A) and performed significantly better on the reading domain than on the math domain of the KTEA achievement assessment (Figure 1B). These relatively lower scores on nonverbal domains of cognitive and achievement assessments are consistent with previous reports of relative strengths in the verbal ability for females with FXS. Relative strengths include acquired knowledge, long-term memory for verbal information, and simultaneous processing [10,41]. Although verbal ability represents a cognitive strength, average verbal scores for girls with FXS remain within the low average range when compared to their age-matched peers. One hypothesis put forward to explain this discrepancy is that the social demands of the language environment for children with FXS (coordination of syntax, semantics, conversational pragmatics, and eye contact) promotes AAA symptoms, which then challenge proper regulation of verbal responses [23]. Thus, it may be important to focus on the development and management of AAA symptoms in girls with FXS to optimize their cognitive as well as social-emotional outcomes.

Although the data showed no significant difference between participant scores on the cognitive assessment and the achievement assessment in our preliminary cohort (Figure 2A,B), it is often assumed that cognitive ability determines achievement. However, children with FXS frequently outperform predictions of academic achievement based on cognitive test scores [23]. One proposed explanation for the discrepancy between achievement and cognitive scores among females with FXS is a relative strength in long-term memory and the effect of repeated exposure to academic material at school [42]. Prior research has also suggested that individuals with FXS have more difficulty processing highly novel information than learning facts and school-related skills [43]. Since intelligence tests and cognitive assessments are intended to be novel and unfamiliar, deficits in flexible thinking may particularly affect performance on these types of tests. These insights may be important for facilitating successful learning outcomes for girls with FXS who exhibit a wide range of learning difficulties. 

### 5.2. Adaptive Behavior Outcomes

Correlations between cognitive scores and adaptive behavior in our preliminary cohort suggest a significant association between nonverbal abilities and overall adaptive behavior. (Figure 3A,B). Given the relative cognitive strength in the verbal ability of individuals with FXS [10,19,20,21,22], it is particularly concerning that these strengths do not appear to be translated readily to outcomes in functional skills. 

Our preliminary results also suggest a negative correlation between social skills and adaptive behavior (Figure 4), suggesting that aberrant social skills and function are associated with challenges in adaptive behavior skills. These findings are consistent with prior research, which has suggested that factors other than cognitive abilities, such as ability to interact appropriately socially [17], autism spectrum disorder type behaviors [30], and symptoms of affective disorders, such as anxiety and depression [4], have significant influence on the development of adaptive behavior skills and functional outcomes for girls with FXS.

## 6. Conclusions

**Figure 5:**
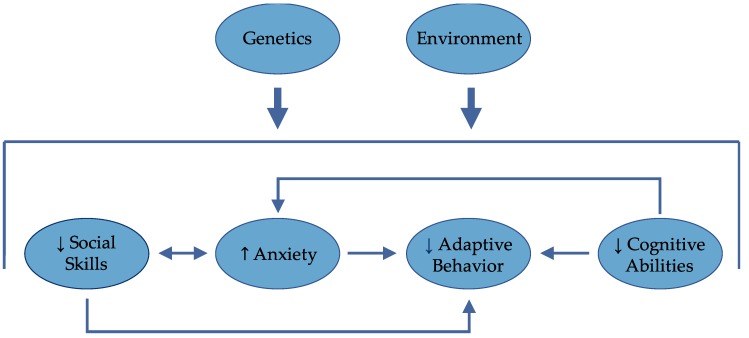
Genes, environment, cognitive, and socio-emotional factors all intersect to determine outcome. Anxiety may be inversely related to social skills, adaptive behavior, and cognitive abilities.

The preliminary data presented here support prior findings that cognitive abilities do not play the only, or even necessarily the primary, role in determining functional outcomes for girls with FXS. Continued research is needed to better understand how functional outcomes are influenced by other critical factors related to academic, home and educational environments, and socio-emotional development (Figure 5). 

Our current study seeks to address the relative paucity of information focusing exclusively on females with the *FMR1* full mutation to elucidate the role of these factors in the development of girls and women with FXS. In particular, a fine-grained examination of gene–environment–behavior associations underlying the development and progression of social skills and symptoms of AAA will provide new information on the degree to which females with FXS experience maladaptive symptoms. We will also gain a better understanding of how and when biological and environmental factors most influence the propensity for these symptoms and the role these symptoms play in determining functional outcomes for this vulnerable population.
